# Quantifying anterior segment vascular changes in thyroid eye disease using optical coherence tomography angiography

**DOI:** 10.1186/s12880-025-01627-y

**Published:** 2025-03-11

**Authors:** Ahmad Masoumi, Pedram Afshar, Hanieh Fakhredin, Hamidreza Ghanbari, Fateme Montazeri, Amirhossein Aghajani, Zahra Montazeriani, Pezhman Pasyar, Haniyeh Zeidabadinejad, Faezeh Moghimpour Bijani, Seyed Mohsen Rafizadeh

**Affiliations:** 1 Ophthalmology Department and Eye Research Center, Farabi Eye Hospital, School of Medicine, Tehran University of Medical Sciences, Tehran, Iran; 2 Department of Internal Medicine, School of Medicine, Shariati Hospital, Tehran University of Medical Sciences, Tehran, Iran; 3 Department of Ophthalmology & Vision Science, Tschannen Eye Institute, University of California, Davis, Sacramento, CA USA; 4 Department of Medical Physics and Biomedical Engineering, Tehran University of Medical Sciences, Tehran, Iran

**Keywords:** TED, AS-OCTA, Conjunctiva, TED activity, CAS

## Abstract

**Purpose:**

Thyroid eye disease (TED) presents challenges in the accurate assessment of disease activity, especially concerning ocular surface manifestations. This study aims to evaluate the potential of anterior segment optical coherence tomography angiography (AS-OCTA) in quantifying vascular changes associated with TED, thereby enhancing understanding of its pathophysiology and aiding in diagnosis and management.

**Methods:**

We conducted a cross-sectional study involving 29 TED patients and 21 healthy controls. Participants underwent comprehensive ophthalmic examination and AS-OCTA imaging of predefined regions of interest (ROI) in the nasal and temporal quadrants. Vascular metrics including vessel density (VD), vessel length density (VLD), vessel diameter index (VDI) and fractal dimension (FD) were analyzed using AS-OCTA software. Disease activity was assessed using clinical activity scores (CAS).

**Results:**

TED patients exhibited increased VD and VLD, particularly in the temporal quadrant, compared to healthy controls. Additionally, TED patients in active disease phases demonstrated larger VDI in the nasal quadrant. Negative correlations were observed between superficial VD and disease activity scores, while positive correlations were found between deep VDI and disease activity.

**Conclusion:**

AS-OCTA demonstrates potential in quantitatively assessing vascular changes in TED, providing valuable insights into its pathophysiology and potential implications for clinical management. Conjunctival vascular parameters might be valuable in grading the TED disease activity in the future.

**Clinical trial number:**

Not applicable.

## Introduction

Thyroid eye disease (TED) is an autoimmune condition with a prevalence of 16 per 100,000 females and 2.9 per 100,000 males [1]. Clinical assessment of TED typically relies on the clinical activity score (CAS) system indicating the intensity of inflammation [1]. The effects of TED can be seen in different parts of the eye. Among its various ocular surface manifestations [2], are conjunctival injection, chemosis, dry eye, keratopathy, and corneal ulcer leading to visual impairment [1].

The pathophysiology of Thyroid Eye Disease (TED) remains partially understood, yet it is known to be closely associated with inflammatory responses and the remodeling of orbital and periorbital soft tissues [1]. These anatomical changes profoundly affect the eye’s vascular network [3]. While research has predominantly focused on the vascular modifications in the retina and choroid [4, 5], TED’s influence indeed reaches beyond the eye’s posterior segment, affecting various aspects of the ocular surface. During the severe inflammatory phase of TED, there is a marked increase in the complexity and blood flow of the bulbar conjunctival vessels [6]. This phenomenon was directly correlated with the disease’s activity levels, as assessed through functional slit lamp biomicroscopy [6], highlighting the broader implications of TED on anterior segments of the eye.

Optical coherence tomography angiography (OCTA) has developed into a cutting-edge diagnostic tool primarily utilized for detecting vascular abnormalities in the posterior segments [7]. Optical coherence tomography has emerged as a non-invasive examination tool, facilitating the diagnosis of various diseases, including systemic conditions, as evidenced by recent studies [8–10]. Recently, its utility has expanded to include evaluations of the anterior segment as well [11]. OCTA provides quantitative assessments of the vasculature networks with details and at various depths [11]. In anterior segment OCTA (AS-OCTA), metrics such as vessel density (VD), vessel length density (VLD), vessel diameter index (VDI), and fractal dimension (FD) are used to measure vascular density and the characteristics of blood flow. These indices are derived from advanced software designed to extract and analyze vascular data. The obtained metrics facilitate comparative analyses across different regions or conditions within the anterior segment, enable monitoring of changes, and assist in diagnostics by comparing against established normative data [12].

In this study, we aim to evaluate alterations in ocular surface microvasculature among patients with TED by employing AS-OCTA, and investigate potential correlations between various vessel parameters and the disease activity. This research represents a significant step towards enhancing our understanding of the anterior segment vascular changes associated with TED. It may offer valuable insights for diagnosing and managing this complex condition.

## Method

The study adhered to the Declaration of Helsinki. Ethics committee approval was obtained from the Tehran University of Medical Sciences. All study participants provided written informed consent before enrollment.

### Study participants

In this cross-sectional study, we evaluated patients clinically diagnosed with TED who presented at the orbit and oculoplastic clinic of Farabi Eye Hospital in Tehran, Iran, over a period of three months, from March to May 2023. Patients with confirmed TED (active or inactive) who had not received prior treatment for the condition were consecutively enrolled in the study. The study excluded individuals younger than 20 or older than 60 years, those with any history of TED-related surgeries (such as orbital decompression, strabismus surgery, or eyelid retraction correction), patients suffering from optic nerve dysfunction or exposure keratopathy, those diagnosed with other ocular surface diseases (e.g., pterygium, allergic conjunctivitis) or having undergone related surgeries, individuals with dry eye disease from non-TED causes (like Sjögren’s disease or severe meibomian gland dysfunction), and finally patients with uncontrolled thyroid function tests.

The control group consisted of healthy individuals aged 29 to 52 years who volunteered to participate and were able to comply with the eye examination protocols. Notably, we included both eyes of each participant from the case and control groups in the study.

All participants underwent a comprehensive ophthalmic examination, including best corrected visual acuity (BCVA), relative afferent pupillary defect (RAPD), measurement of margin-to-reflex distance (MRD) 1 and 2, and proptosis. We assessed disease activity using the CAS, which includes spontaneous retrobulbar pain, pain in vertical eye movement, eyelid erythema, eyelid edema, conjunctival injection, conjunctival chemosis, and caruncle inflammation. A score was considered for each of these criteria.

Based on their CAS scores, patients were divided into two groups: those with a CAS score of 3 or higher were considered to have active TED, whereas those with a score below 3 were classified as having inactive TED.

### Anterior segment optical coherence tomography angiography

All study participants underwent AS-OCTA imaging at the nasal and temporal conjunctival area. Experienced photographers conducted the imaging sessions using the AngioVue OCTA system manufactured by Optovue, CA, USA. The total scan acquisition time of AngioVue OCTA was less than 3 s. The device operated at a central wavelength of 840 nm and had a beam width of 22 μm. It achieved a scanning speed of 70,000 A-scans per second and provided an optical axial resolution of approximately 5 μm. To ensure maximum image resolution, we selected a scan pattern of 3 × 3 mm for each participant. The device acquired 304 × 304 A-scans, capturing two consecutive B-scans at each position to distinguish between static tissues and structures with high signal fluctuation. Blood flow was detected by combining the two images at the exact location using proprietary angiography algorithms and motion correction techniques. The Split Spectrum Amplitude Decorrelation Angiography (SSADA) algorithm in AngioVue enhanced the signal-to-noise ratio and improved flow detection.

### Image acquisition and segmentation

We utilized the Angioretina mode of the AngioVue OCTA system to visualize and quantify the vasculature in the anterior segment of the eye. Our analysis focused on several vascular indexes within predefined regions of interest (ROI). A 3 × 3 mm scan was employed to acquire AS-OCTA images. Measurements were conducted on en face images of the superficial and deep layers in the nasal and temporal regions. OCT images were processed using MATLAB (version 2021b). To reduce artifacts and enhance vessel contrast, contrast-limited adaptive histogram equalization (CLAHE) and median filtering were applied to the grayscale images. An expert manually identified the region of interest (ROI) and the limbus edge. The Otsu thresholding method was then used to binarize the images, effectively isolating the vessels by selecting a threshold that minimizes intra-class variance between foreground and background pixels. Finally, Vessel Density (VD), Vessel Length Density (VLD), Vessel Diameter Index (VDI), and Fractal Dimension (FD) were calculated within the target region.

To ensure the accuracy of segmentation, we performed a validation step involving manual correction of the identified ROIs by the same expert. Furthermore, interobserver and intraobserver variability for OCTA measurements were assessed using a subset of images. The interobserver variability was evaluated by having a second independent observer repeat the segmentation process, while intraobserver variability was determined by having the same observer repeat the measurements after a set interval. These assessments highlighted a high degree of consistency in our measurements, supporting the reliability of our image processing methodologies.

1. **Vessel Density (VD)**: This index represents the proportion of the ROI occupied by blood vessels, offering insights into the vessel density within that area. The formula for VD is given by:

$$\:VD=\frac{Total\:area\:of\:vessels\:in\:ROI\:}{Total\:area\:of\:ROI}$$

2. **Vessel Length Density (VLD)**: VLD measures the cumulative length of blood vessels per unit area in a specific ROI. This index provides information on the vessel length distribution within a region. It is calculated as follows:

$$\:VLD=\frac{Total\:length\:of\:vessels\:in\:ROI\:}{Total\:area\:of\:ROI}$$

3. **Vessel Diameter Index (VDI)**: VDI calculates the average diameter or caliber of blood vessels within a particular ROI, offering details on vessel thickness. The formula for VDI is:

$$\:VDI=\frac{Total\:cross-sectional\:area\:of\:vessels\:in\:ROI\:}{Total\:length\:of\:vessels\:in\:ROI}$$

4.**Fractal Dimension (FD)**: The Fractal Dimension quantifies the complexity and geometric structure of blood vessel networks within the region of interest (ROI). It provides insights into the spatial distribution and branching patterns of the vasculature. Mathematically, FD can be expressedas:

$$\:FD=-\frac{d\left(\text{log}\left(N\right)\right)\:}{d\left(\text{log}\left(e\right)\right)}$$

where “N” is the number of boxes containing part of the vessel structure, and “e” is the size of the boxes.

The vascular parameters of each scanned image were assessed at two distinct depths: (1) Superficial, from conjunctival epithelium to a depth of 200 μm. (2) Deep, from a depth of 200 μm to a depth of 1000 μm, indicating the intrascleral layer. The conjunctival area within the image was manually identified by aligning it with the inner limiting membrane as indicated by the device. Further, each image was subdivided into temporal and nasal quadrants for detailed analysis.

### Statistical analysis

Statistical analyses were performed using IBM SPSS Statistics software, version 27 (IBM Corp, New York, USA) and Python (Python Software Foundation, https://www.python.org/). Descriptive statistics were calculated for the study variables, including the mean and standard deviation (SD). Given the inclusion of data from both eyes of the same individuals, we employed a generalized estimating equation model with robust estimators to compare the case and control groups while controlling for potential age and gender effects. In this model, the vascular features were dependent variables, while age, gender, and grouping (case vs. control) were considered independent variables. We excluded the missing data from the analysis. Additionally, Sidak correction was used in multiple comparisons. Statistical significance was defined as a p-value less than 0.05.

It is important to note that OCTA imaging can be susceptible to various artifacts, including motion artifacts and projection artifacts, which may influence the accuracy of vascular measurements. Additionally, inter-device variability can occur, particularly when using different OCTA systems, potentially affecting comparative analyses. Future studies should focus on standardizing imaging protocols and assessing the consistency of results across different OCTA devices to validate our findings further.

## Results

### Characteristics of subjects

We enrolled 58 eyes from 29 patients with TED (41.3% female) and 42 eyes from 21 healthy control participants (38.1% female). The mean (± SD) age was 46.2 ± 10.8 years in the patient group, and 32.7 ± 6.6 years in the control group (*p* < 0.001). The baseline characteristics of the study participants are summarized in Table 1.

**Table 1:** Demographics and clinical features of participants, including both TED patients and healthy controls

Demographics				
	Case (Included *N*= 29)	Control (Included *N*= 21)	*P* Value^*^	
Eye, No	58	42	NA	
Female (%)	24 (41.3)	26 (61.9)	0.06	
Age, year (± SD)	46.2 (± 10.8)	32.7 (± 6.6)	<0.001	
**TED Characteristics**,** mean (± SD)**				
	**Active**	**Inactive**	P Value**	**Adjusted P Value** ^**^
Eye, No (%)	32 (55.1)	26 (44.8)	NA	NA
TED Duration, month	26.8 (38.1)	46.4 (48.6)	0.25	0.47
Proptosis, mm	24.6 (3)	23 (2.5)	0.09	0.16
MRD1, mm	6.9 (2)	6.9 (2)	0.97	0.11
MRD2, mm	6.9 (1.1)	7 (0.9)	0.79	0.4
Visual acuity, LogMAR	0.15 (0.2)	0.06 (0.1)	0.18	0.02

*Chi square test for gender and t-test for age. **Generalized estimation equation (adjusted for age and gender)

Abbreviations: TED=Thyroid eye disease, SD=Standard deviation, MRD=Marginal reflex distance, LogMAR= Logarithm of the Minimum Angle of Resolution

### Vascular features

**Fig. 1:**
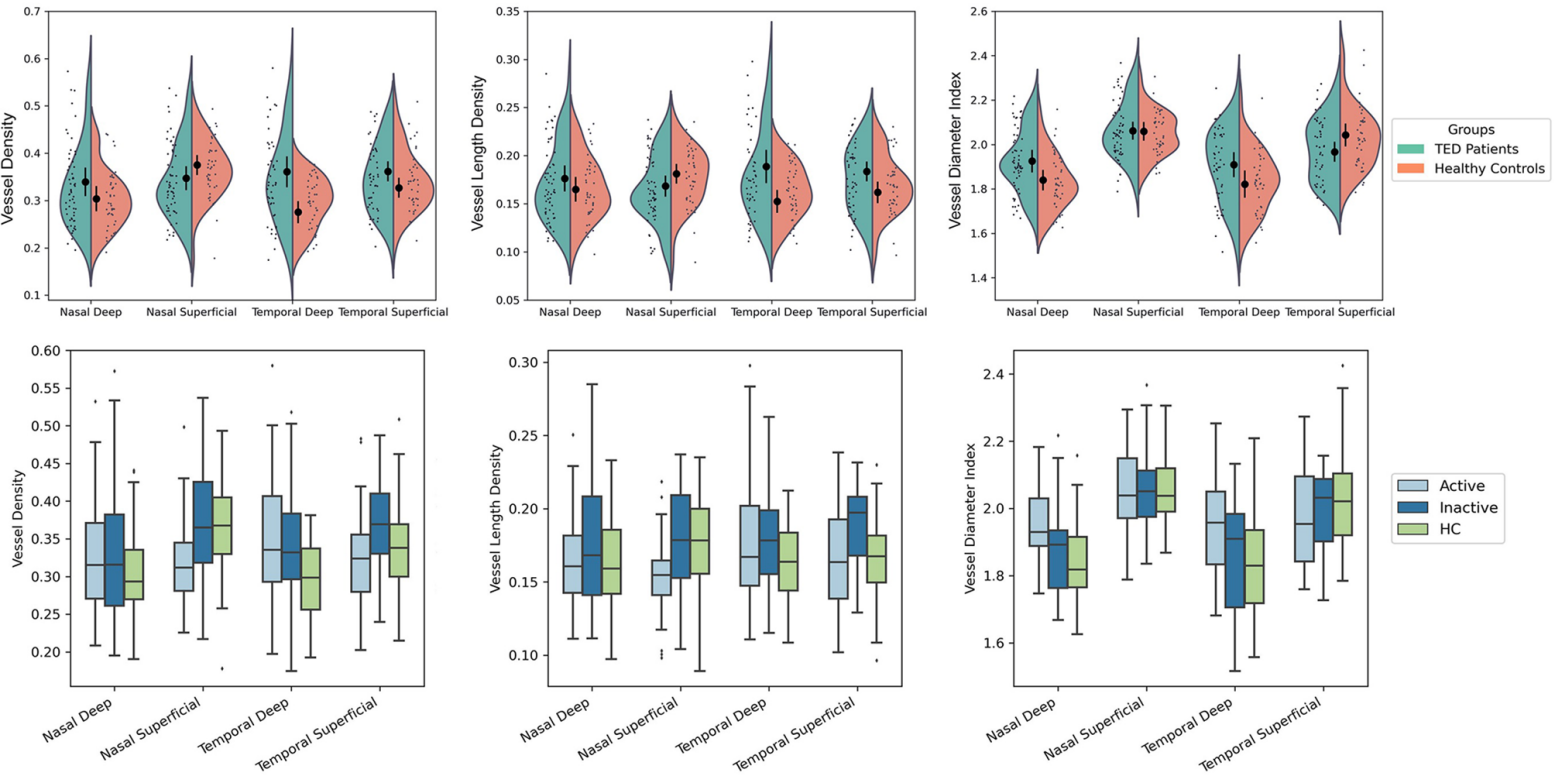
Split violin plot (upper) showing the distribution of different vascular parameters among TED patients and healthy controls. The black circles with associated lines represent each parameter’s adjusted mean and 95% confidence interval. The boxplot (lower) displays the vascular measurements among active, inactive, and healthy controls (HC)

Table 2 presents a comparison of vascular features, including VD, VLD, VDI, and FD after adjusting for age and gender differences between the case and control eyes. VD was greater in TED patients in temporal deep (adjusted mean difference (aMD) 0.08, 95% confidence interval (CI) [0.03–0.1], *p* < 0.001) and temporal superficial quadrants (aMD 0.03, 95% CI [0.005–0.06], *p* = 0.02). TED patients also had greater VLD in temporal regions (deep area: aMD 0.03, 95% CI [0.01–0.06], *p* = 0.005; superficial area: aMD 0.02, 95% CI [0.006–0.03], *p* = 0.008). VDI was greater in the nasal deep quadrant among TED patients (aMD 0.08, 95% CI [0.01–0.1], *p* = 0.01), while temporal superficial VDI was smaller among these patients compared to healthy controls (aMD − 0.07, 95% CI [-0.14- -0.008], *p* = 0.02). No difference in FD was observed between the two groups (Fig. 1).

**Table 2:** Summary of generalized estimation equation comparing vascular features between TED eyes and controls ^a^

Characteristics	Region	Layer depth	Mean ± SD		Adjusted mean differences	95% CI	*P*-value	
			TED eyes (*n*= 58)	Control eyes (*n*= 42)				
Vessel density	Nasal	Superficial	0.341 ± 0.07	0.364 ± 0.06	-0.028	(-0.064) - (0.008)	0.125	
		Deep	0.330 ± 0.08	0.302 ± 0.06	0.036	(-0.007) - (0.078)	0.101	
	Temporal	Superficial	0.346 ± 0.07	0.339 ± 0.06	0.035	(0.005) - (0.064)	0.021	
		Deep	0.344 ± 0.09	0.296 ± 0.05	0.085	(0.038) - (0.133)	<0.001	
Vessel length density	Nasal	Superficial	0.165 ± 0.03	0.177 ± 0.03	-0.013	(-0.029) - (0.004)	0.131	
		Deep	0.171 ± 0.04	0.165 ± 0.03	0.011	(-0.009) - (0.031)	0.272	
	Temporal	Superficial	0.175 ± 0.03	0.168 ± 0.03	0.022	(0.006) - (0.038)	0.008	
		Deep	0.180 ± 0.04	0.163 ± 0.02	0.036	(0.011) - (0.062)	0.005	
Vessel diameter index	Nasal	Superficial	2.06 ± 0.12	2.05 ± 0.09	0.003	(-0.062) - (0.067)	0.932	
		Deep	1.92 ± 0.13	1.83 ± 0.11	0.085	(0.014) - (0.157)	0.019	
	Temporal	Superficial	1.97 ± 0.14	2.02 ± 0.13	-0.076	(-0.144) - (-0.008)	0.028	
		Deep	1.90 ± 0.16	1.83 ± 0.13	0.087	(-0.009) - (0.183)	0.07	
Fractal dimension	Nasal	Superficial	1.85 ± 0.031	1.85 ± 0.033	-0.005	(-0.023) - (0.012)	0.54	
		Deep	1.85 ± 0.036	1.85 ± 0.032	-0.013	(-0.030) - (0.003)	0.10	
	Temporal	Superficial	1.83 ± 0.04	1.84 ± 0.03	-0.0003	(-0.015) - (0.016)	0.97	
		Deep	1.83 ± 0.04	1.83 ± 0.03	-0.012	(-0.029) - (0.004)	0.15	

^a^ Adjusted for age and gender. Abbreviations: CI=Confidence interval, SD=Standard deviation, TED=Thyroid eye disease.

**Fig. 2:**
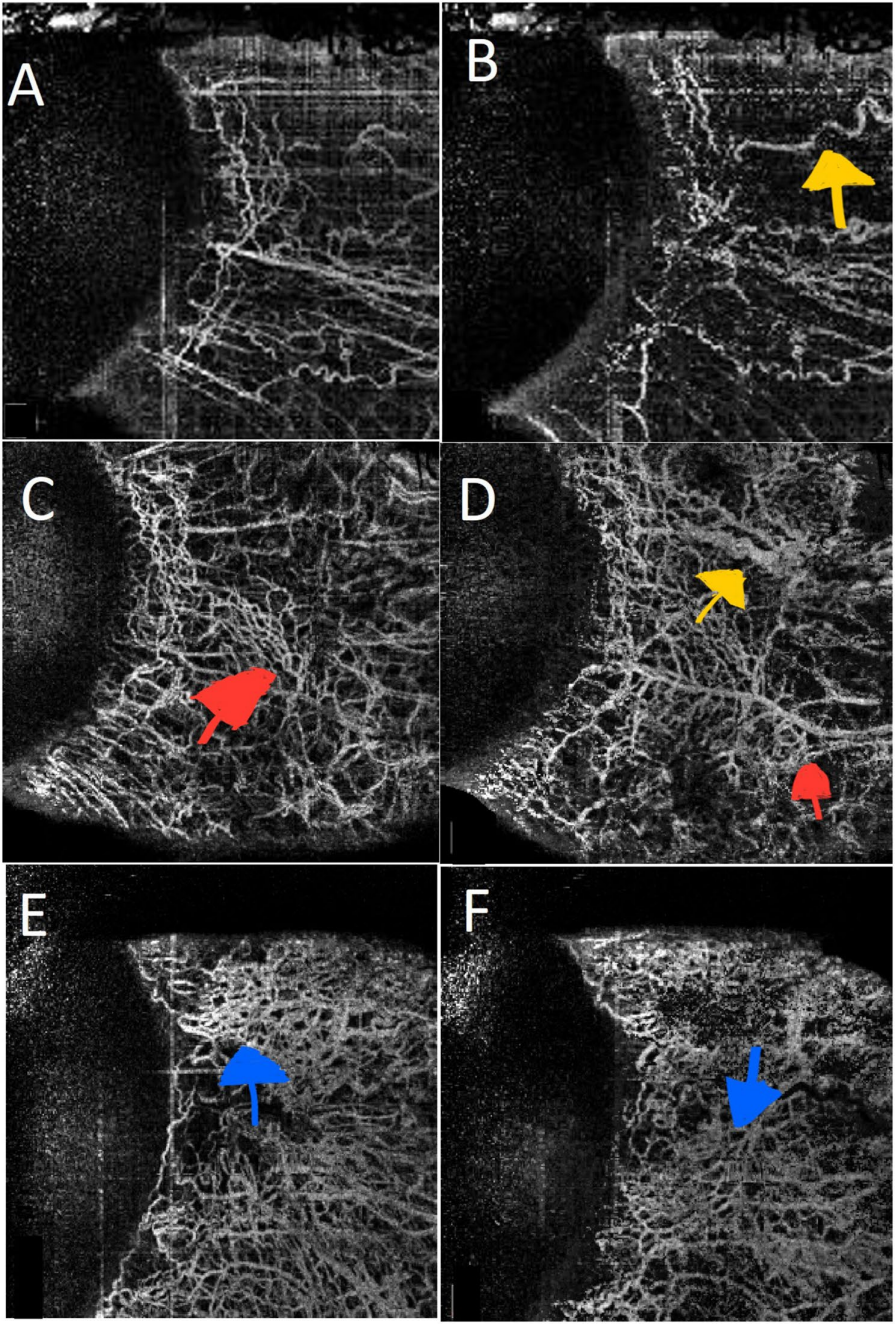
Optical coherence tomography angiography (OCTA) of the superficial conjunctival vessels in a normal eye showing the normal centrifugal pattern of superficial conjunctival vasculature (**A**). Straight deep scleral vessels (yellow arrowhead) are seen traversing the OCTA of the deep vascular plexus in a normal eye (**B**). Increased vascular density (red arrowheads) is observed in a patient with inactive TED in the OCTA of superficial (**C**) and deep (**D**) vasculature of the ocular surface. Dilated scleral vessels (yellow arrowhead) are visible in the OCTA of the deep vasculature in a patient with inactive TED (**D**). More haphazard vessels (blue arrowheads) and increased vessel diameter are observed in the superficial (**E**) and deep (**F**) ocular surface vessels in a patient with active TED

The vascular parameters that showed a significant difference between active and inactive TED eyes included the VDI, which was notably greater in the active form of the disease in the nasal deep quadrant (aMD 0.1, 95% CI [0.02–0.19], *p* = 0.009). Additionally, the FD in the temporal region was greater in the inactive group for both the deep and superficial layers (deep area: aMD 0.02, 95% CI [-0.049–0.0009], *p* = 0.04; superficial area: aMD 0.02, 95% CI [-0.0535–0.0009], *p* = 0.04 ) (Table 3). Figure 2 presents representive OCTA images of a normal eye, an eye with active TED, and an eye with inative TED.

**Table 3:** Summary of generalized estimation equation comparing vascular features between active and inactive TED eyes ^a^

Characteristics	Region	Layer depth	Mean ± SD		Adjusted mean differences	95% CI	*P*-value
			Active TED eyes (*n*= 32)	Inactive TED eyes (*n*= 26)			
Vessel density	Nasal	Superficial	0.318 ± 0.06	0.369 ± 0.08	0.03287	(-0.01) - (0.08)	0.348
		Deep	0.326 ± 0.07	0.334 ± 0.09	-0.02138	(-0.08) - (0.04)	0.838
	Temporal	Superficial	0.326 ± 0.07	0.371 ± 0.06	0.02354	(-0.02) - (0.07)	0.645
		Deep	0.349 ± 0.09	0.338 ± 0.08	-0.03091	(-0.1) - (0.03)	0.639
Vessel length density	Nasal	Superficial	0.154 ± 0.03	0.178 ± 0.03	0.01621	(-0.005) - (0.03)	0.207
		Deep	0.166 ± 0.03	0.176 ± 0.04	-0.00185	(-0.03) - (0.2)	0.999
	Temporal	Superficial	0.166 ± 0.03	0.187 ± 0.03	0.01109	(-0.01) - (0.03)	0.630
		Deep	0.179 ± 0.05	0.181 ± 0.04	-0.00847	(-0.04) - (0.02)	0.916
Vessel diameter index	Nasal	Superficial	2.062 ± 0.12	2.061 ± 0.13	-0.01099	(-0.09) - (0.07)	0.986
		Deep	1.955 ± 0.11	1.884 ± 0.15	-0.10737	(-0.19) - (-0.02)	0.009
	Temporal	Superficial	1.971 ± 0.15	1.978 ± 0.12	-0.01000	(-0.13) - (0.11)	0.997
		Deep	1.946 ± 0.15	1.86 ± 0.17	-0.07759	(-0.19) - (0.03)	0.295
Fractal dimension	Nasal	Superficial	1.85 ± 0.031	1.85 ± 0.033	-0.013	(-0.035) - (0.008)	0.22
		Deep	1.84 ± 0.037	1.85 ± 0.036	-0.009	(-0.029) - (0.010)	0.36
	Temporal	Superficial	1.82 ± 0.053	1.85 ± 0.028	-0.027	(-0.053) - (-0.0009)	0.04
		Deep	1.83 ± 0.043	1.84 ± 0.041	-0.025	(-0.049) - (-0.0009)	0.04

### Vascular features and disease activity

In nasal and temporal superficial layers, the results from linear regression analyses demonstrated a significant negative correlation between CAS scores and VD (nasal: -0.01, 95%CI [-0.02 to -0.004], *p* = 0.003; temporal: -0.01, 95%CI [-0.02 to -0.006], *p* = 0.001) as well as VLD (nasal: -0.006, 95%CI [-0.009 to -0.003], *p* < 0.001; temporal: -0.007, 95%CI [-0.01 to -0.002], *p* = 0.003). Conversely, there was a positive correlation between CAS scores and VDI in the nasal deep quadrant, indicating a significant increase with higher CAS scores (0.03, 95% CI [0.01 to 0.04], *p* < 0.001). Additionally, FD results showed a negative correlation in the temporal quadrant in both deep and superficial layers (superficial: -0.009, 95%CI [-0.015 to -0.001], *p* = 0.01; deep: -0.007, 95%CI [-0.013 to -0.0008], *p* = 0.03). (Table 4)

**Table 4:** Association of vascular features and CAS score among TED eyes ^a^

Characteristics	Region	Layer depth	Adjusted coefficient	95% CI	*P*-value
Vessel density	Nasal	Superficial	-0.013	(-0.02) - (-0.004)	0.003
		Deep	0.004	(-0.01) - (0.01)	0.599
	Temporal	Superficial	-0.015	(-0.023) - (-0.006)	0.001
		Deep	-0.001	(-0.014) - (0.013)	0.937
Vessel length density	Nasal	Superficial	-0.006	(-0.009) - (-0.003)	<0.001
		Deep	0.000	(-0.007) - (0.006)	0.893
	Temporal	Superficial	-0.007	(-0.011) - (-0.002)	0.003
		Deep	-0.001	(-0.008) - (0.006)	0.77
Vessel diameter index	Nasal	Superficial	0.002	(-0.01) - (0.01)	0.83
		Deep	0.031	(0.01) - (0.04)	<0.001
	Temporal	Superficial	-0.005	(-0.029) - (0.019)	0.685
		Deep	0.013	(-0.004) - (0.013)	0.142
Fractal dimension	Nasal	Superficial	-0.003	(-0.008) - (0.002)	0.18
		Deep	-0.004	(-0.01) - (0.001)	0.13
	Temporal	Superficial	-0.009	(-0.015) - (-0.001)	0.01
		Deep	-0.007	(-0.013) - (-0.0008)	0.03

^a^ Adjusted for age and gender. Abbreviations: CAS=Clinical activity score, CI=Confidence interval, SD=Standard deviation, TED=Thyroid eye disease.

## Discussion

TED significantly affects the ocular surface, necessitating thorough clinical evaluations [2]. The grading of TED includes assessing both its activity, which involves inflammation and disease progression, and severity, which encompasses the extent of involvement and functional deficits [1]. Treatment strategies vary based on these factors and often require a multidisciplinary approach to manage the disease effectively and prevent further progression [13]. For instance, immunosuppressants are crucial in moderate to severe active cases, while surgical interventions are more appropriate during the inactive phase [1]. CAS is commonly used to evaluate TED activity, but it struggles to quantify subtle changes, especially in deeper ocular structures, due to its subjective nature [14]. The subjectivity leads to significant interrater and intrarater variability among investigators [15]. Additionally, CAS often fails to accurately report disease activity in patients with darker complexions [16]. As a result, many researchers are seeking more objective methods to assess TED activity. Measuring conjunctival vascular parameters using AS-OCTA might prove useful for this purpose.

This study introduces an innovative use of AS-OCTA to quantitatively assess vascular changes in TED patients. Our results indicate increased vessel density in the temporal quadrant and larger deep vessel diameters in the nasal quadrant, particularly during the active stage of TED. A positive correlation between deep vessel diameter and CAS as well as a negative correlation in superficial vessel density, were observed.

A promising framework for comprehending TED-related alterations stems from The Cone Model [3]. It proposes that the enlargement of the orbital cone, triggered by the accumulation of glycosaminoglycans, leads to the expansion of the rectus muscles and displacement of fat. These changes consequently escalate intraconal pressure during TED [3]. This pressure can potentially impede venous drainage to the cavernous sinus. Physiologically, conjunctival venules drain anteriorly into the limbal venous circle to reach episcleral collecting and ophthalmic veins and drain into the cavernous sinus [17]. However, flow disturbance through the superior ophthalmic vein in TED can cause reversal conjunctival venous flow into the posterior tarsal and eyelid circulations [3]. Our results support the Cone Model by showing increased deep nasal quadrant VD in patients with active TED, suggesting posterior venous drainage disturbance. The nasal quadrant’s significant involvement is likely due to its proximity to the superior ophthalmic vein and the common involvement of inferior and medial rectus muscles in TED [18].

Our findings highlight a significant difference in orbital congestion between active and not active TED(Table 3). The larger deep VD in the nasal quadrant during active TED and the positive correlation between CAS and VD in this area aligns with the Cone Model’s predictions, indicating increased cone pressure correlates with more inflammation in TED [3]. Studies utilizing Doppler imaging techniques also confirmed that the reduced or reversed outflow in the superior ophthalmic vein contributed to increased congestion in the congestive type of TED [19, 20].

Our study acknowledges that while assessing the superior ophthalmic vein is crucial in understanding TED, this method alone might not fully capture the hemodynamic changes in orbital vessels. Systemic factors and inflammation can also influence vascular parameters [2]. Supporting this perspective, a previous study using functional slit lamp biomicroscopy demonstrated increased vascular density and diameter in the bulbar conjunctiva of TED patients [6]. This study found significant correlations between these morphological features and CAS. Our findings contribute to this understanding by observing a higher VD in the temporal quadrant of TED patients. This finding is particularly relevant regarding TED’s propensity to induce long-term inflammation in the orbit. During the inflammatory phase of TED, there is an increase in vascular endothelial growth factor and its receptors, potentially resulting in neovascularization and increase VD, especially in the acute active phase of the disease [21]. While our findings are consistent with these mechanisms, it’s notable that the disparities between active and not active phases of TED in our study did not attain statistical significance.

Furthermore, our study discovered a negative correlation between superficial VD and CAS. This could be linked to the chronic progression of TED, which often results in persistent mechanical impairments like lid retraction and proptosis [22]. Such impairments can lead to dry eye syndrome, which can cause changes in the vascular indices of the anterior segment [23, 24]. However, in recent studies, dry eye has also been reported in the early stages of TED [25, 26]. This aspect underscores the complex interplay between the disease’s course and its ocular manifestations, highlighting the need for a nuanced approach in the clinical assessment and management of TED.

In this study, we demonstrated that vascular indices in specific quadrants of the anterior segment are elevated in TED patients compared to normal subjects. Among patients with TED, the rise in VD is particularly notable in the superficial temporal and deep temporal regions, while those with active TED exhibit increased vascular diameter index primarily in the deep nasal region. Clinical manifestations observed in active TED cases, such as the presence of a clear zone without hyperemia at the insertion site of rectus muscles, along with hypertrophy and deep inflammation in the plica semilunaris and caruncle (medially), corroborate our study findings.

We believe AS-OCTA might be a useful adjunctive tool for clinicians to detect early signs of TED and evaluate the activity of this disease, thereby aiding in its management. TED can lead to vision threatening complications if it not treated promptly, including exposure keratopathy and compressive optic neuropathy [27]. More prospective studies are needed to evaluate the utility of AS-OCTA in early detection of these complication and their timely treatment.

In recent studies, the application of advanced machine learning techniques, such as the Multi-style Spatial Attention module proposed by Xiao et al. (2024), has demonstrated enhanced capabilities in classifying cataracts using AS-OCT images [28]. This approach leverages clinical context to improve diagnostic accuracy, which may provide a valuable methodological framework for our investigations into TED-related vascular changes.

The need for efficient algorithms in medical imaging is underscored by the work of He et al. (2024), who developed a lightweight network for retinal layer segmentation that combines local and global reasoning [29]. Such methodologies could be adapted to optimize AS-OCTA imaging techniques, enhancing the robustness of our vascular assessments in TED patients.

The regional context-based recalibration network introduced by Zhang et al. (2024) emphasizes the importance of utilizing clinical prior information to improve feature extraction in cataract recognition tasks [30]. Similarly, our study could benefit from employing such recalibration techniques to enhance the precision of vascular metrics in AS-OCTA, particularly in distinguishing between disease activity levels in TED patients.

Challenges in low-contrast image segmentation, as highlighted by Zhang et al. (2024), demonstrate the necessity for innovative approaches that combine both convolutional and transformer architectures [31]. This insight may inform future improvements in AS-OCTA processing methods, particularly in the context of visualizing and quantifying subtle vascular changes in TED, which can be exacerbated by low-contrast imaging conditions.

We acknowledge the limitations of the small sample size and the use of OCTA software designed for posterior segments, which may introduce more artifacts in anterior segment evaluation. Despite limitations, this study concludes that nasal intrascleral VD measured by AS-OCTA is a significant potential indicator of TED activity. It highlights the need for improving AS-OCTA techniques as a promising tool for evaluating vascular changes in the anterior segment of the eye among TED patients. Further comprehensive studies involving a larger cohort are warranted to validate the current findings and enhance our understanding of the pathophysiology of TED. Recent advancements in dynamic contrastive learning frameworks emphasize the importance of adaptively selecting informative pixels during training [32]. By focusing on pixel difficulty, these methods can significantly enhance the segmentation accuracy of complex medical images, including those obtained from AS-OCTA. Incorporating semi-supervised learning techniques can also provide a pathway to improve the performance of our segmentation analyses [33]. By leveraging unlabelled data alongside a limited set of annotated images, we can enhance the robustness of AS-OCTA metrics in assessing changes associated with thyroid eye disease. Furthermore, addressing the challenge of inconsistent labeling is crucial for ensuring reliable segmentation outcomes. Techniques such as those described by Chen et al. (2025), which utilize cross-image matching to guide segmentation, can help mitigate the effects of annotation discrepancies in our AS-OCTA analyses [34].

## Conclusion

Our study demonstrates that Anterior Segment Optical Coherence Tomography Angiography (AS-OCTA) is a promising tool for quantitatively assessing vascular alterations in patients with Thyroid Eye Disease (TED). We found that TED patients exhibited significant increases in vessel density (VD) and vessel length density (VLD) in the temporal quadrant and a larger vessel diameter index (VDI) in the nasal quadrant during active disease phases. These findings suggest that AS-OCTA can effectively capture the vascular changes associated with TED, offering a more objective and quantifiable method to assess disease activity compared to traditional clinical evaluation methods such as the Clinical Activity Score (CAS).

Furthermore, our study highlights the potential of conjunctival vascular parameters, particularly in the anterior segment, as valuable biomarkers for grading disease activity in TED. The observed negative correlation between superficial VD and CAS, as well as the positive correlation between deep VDI and CAS, underscores the complexity of ocular surface changes in TED and their relationship with disease progression. These insights not only enhance our understanding of TED’s pathophysiology but also open avenues for using AS-OCTA in routine clinical practice to monitor disease activity and response to treatment.

In conclusion, AS-OCTA may serve as a crucial adjunctive tool for clinicians in the early detection and management of TED, ultimately aiding in preventing severe complications such as exposure keratopathy and compressive optic neuropathy. Future research should focus on larger, multicentric studies to validate these findings and further explore the role of AS-OCTA in monitoring the therapeutic effects and long-term outcomes in TED patients. Given the cross-sectional nature of our study, longitudinal investigations are essential to validate these findings over time and assess the progression of vascular changes in TED patients.

## Data Availability

The datasets used and/or analysed during the current study are available from the corresponding author on reasonable request.
